# Carbuncle and Furuncle

**Published:** 1891-05-16

**Authors:** 


### The Practitioner's Mirror and Retrospect.

[*The Editor will be glad to receive offers of co-operation and contributions from members of the profession. All letters should be addressed to* The Editor, The Lodge, Porchester Square, London, W.]

### MEDICINE.

### CARBUNCLE AND FURUNCLE.

But little advance has been made in the way of clearing up the pathology of carbuncle. We know that the streptococcus pyogenes aureus, and sometimes the streptococcus pyogenes albus, are to be found in the pus of carbuncle as well as in furuncles. And if we are correct in regarding these micro-organisms as the essential cause of furuncle, we must be consistent in attributing to them an important rôle in the formation of carbuncle. But though it has been customary to speak of carbuncle as simply multiple furuncle, it is well known that there is a greater clinical distinction than pathologists have allowed, and that the symptoms associated with a single carbuncle are far more severe than usually found in cases of boils. If we, on the other hand, seek for new methods of treatment of carbuncle, we find that the literature is less disappointing. The use of antiseptic applications find favour with many observers. Thus, Professor Verneuil, at a meeting of the Academy of Medicine, Paris, stated that in 1883, in a severe case of carbuncle, he directed that a carbolised spray be frequently applied in order to thoroughly disinfect the parts previous to operative interference. He was astonished at finding so much improvement under the use of the carbolised spray that surgical interference became unnecessary. From that time he employed simply the carbolised spray in all cases of carbuncle coming under his treatment, and with better results than he had ever observed before, for whether the carbuncles were large or small, painful or indolent, whether they had sloughed, been opened by incision, or whether the skin was unbroken, he always employed this method of treatment and never failed to get excellent results. He uses a steam spray with a two per cent. solution of carbolic acid, placing the apparatus about ten inches from the affected parts, and regulating the intensity of the spray according to the feelings of the patient. The spray is applied three or four times daily for about twenty minutes, and in the intervals the parts are dressed with lint dipped in a two per cent. solution of carbolic acid.

Sibthorpe, of Madras, irrigates a carbuncle at frequent intervals with warm perchloride of mercury solution, and between the irrigations keeps the part covered with a wet pad of the solution. In two cases reported by him, scraping was also employed, and although in one of these the carbuncle was as large as a soup plate the patient recovered. Another Indian practitioner has reported excellent results from the application of a one in 40 solution of carbolic acid or a one in 2,000 solution of perchloride of mercury.

Veiel, of Cannstatt, has summarised the main indications in the treatment of furunculosis thus: I. Destroy the micrococci by germicidal applications before necrosis of the tissues has been produced. II. Should necrosis have commenced, the separation of the necrosed cone must be hastened as much as possible. III. Prevent the formation of fresh furuncles by infection from the earlier ones. IV. Render the constitution able to resist the invading micrococci as far as possible. The first indication has rarely to be met as the case has generally gone too far before brought under notice. For the second, the old-fashioned poultice is commended; and the poultice and the neighbouring tissues should be brushed with a one per cent. solution of perchloride of mercury, or if that causes too much irritation, with a four per cent. solution of boracic acid. For the night the boils are to be covered with an ointment composed of oxide of zinc and vaseline in equal parts, with four per cent. of boracic acid. The furuncles should not be opened with the knife, unless they are exceptionally slow in developing. Arsenic and sulphide of calcium are regarded as quite useless by the author. This, however, is contrary to the experience of many observers. Sulphide of calcium in doses of $\frac{1}{30}$ to $\frac{1}{10}$ grain three or four times daily generally exercises a rapid and beneficial action in the treatment of furuncle and carbuncle alike. The sulphide of arsenic seems to combine the therapeutical action of these remedies, and is perhaps more effectual than either arsenic or sulphide of calcium alone.

